# Hospitalization of high and low inpatient service users before and after enrollment into Assertive Community Treatment teams: a naturalistic observational study

**DOI:** 10.1186/s13033-016-0052-z

**Published:** 2016-02-29

**Authors:** Hanne Clausen, Anne Landheim, Sigrun Odden, Jūratė Šaltytė Benth, Kristin Sverdvik Heiervang, Hanne Kilen Stuen, Helen Killaspy, Torleif Ruud

**Affiliations:** Department of Research and Development, Mental Health Services, Akershus University Hospital, Lørenskog, Norway; Institute of Clinical Medicine, University of Oslo, Oslo, Norway; National Centre for Dual Diagnosis, Innlandet Hospital Trust, Brumunddal, Norway; Addiction Research, University of Oslo, Oslo, Norway; HØKH Research Centre, Akershus University Hospital, Lørenskog, Norway; Division of Psychiatry, University College London, London, UK

**Keywords:** Assertive community treatment, Hospitalization, High inpatient service use, Appropriate services

## Abstract

**Background:**

Assertive Community Treatment (ACT) is more successful in reducing hospitalization when baseline use is high. However, with a growing recovery-focus, ACT may be useful for people with severe mental illness who are difficult to engage but not high users of inpatient services. This study investigated hospitalization 2 years before and 2 years after ACT enrollment amongst patients both with and without high inpatient services use before enrollment into ACT.

**Methods:**

This naturalistic observational study included 142 patients from 12 different ACT teams throughout Norway. Of these, 74 (52 %) were high users of inpatient services before ACT. The teams assessed the patients upon enrollment using clinician-rated and self-reported questionnaires. Hospitalization data from 2 years before and 2 years after enrollment into ACT were obtained from the Norwegian Patient Registry. Linear mixed models were used to assess changes in hospitalization and to explore associations between these changes and patient characteristics.

**Results:**

When the participants enrolled into the ACT teams, high users of inpatient care were younger, more often living alone and more often subject to involuntary outpatient treatment than low users. The participants spent significantly fewer days in hospital during the 2 years of ACT follow-up compared to the 2 years before enrollment. The reduction was more evident amongst high users, whereas low users had an initial increase in inpatient days in the first year of ACT and a subsequent decrease in the second year. More severe negative symptoms and previous high use of inpatient care were associated with a reduction in both total and involuntary inpatient days. Additionally, a reduction in involuntary inpatient days was associated with being subject to involuntary outpatient treatment upon enrollment into ACT.

**Conclusion:**

The findings in this study may suggest that ACT contributes to more appropriate use of inpatient care, possibly by reducing the presumably avoidable hospitalization of high users and increasing the presumably needed inpatient care of low users.

## Background

Hospitalization is considered a proxy for symptom relapse in schizophrenia and is a frequently used measure of treatment effectiveness in studies investigating services that target this population [1, 2]. Assertive Community Treatment (ACT) is a well-documented model of community based care that provides outreach services to people with severe mental illness (schizophrenia, other psychotic disorders or severe bipolar disorder), co-morbidities and poor functioning [3–5]. ACT has been found to successfully reduce hospitalization amongst people with severe mental illness such as schizophrenia and bipolar affective disorder, who have difficulties engaging with standard care and experience recurrent cycles of relapse and readmission to mental hospitals [6, 7]. The ACT approach provides more flexible and intensive support, including evidence-based and individually tailored services in the community, than generic mental health services [7, 8].

One of the primary aims of ACT is to reduce the extent and associated cost of inpatient service use [3] but the setting in which ACT is more appropriate for implementation and effective is debatable [9, 10]. ACT has a superior effect on hospitalization over standard mental health services where there is less overlap between the support delivered by services [11, 12] and when it is focused on high users of inpatient care [6, 12]. Conversely, patients with low inpatient service use prior to ACT may experience an increase in hospitalization once under the care of ACT [13, 14]. Indeed, Mortimer and colleagues concluded that ACT is appropriate for patients with a range of needs, not only those who are high users of inpatient services [15].

ACT is intended for persons with mental illness with the most severe symptoms and disabilities who are prone to frequent or long admissions. This includes patients with poor community functioning who are not successfully engaged by less intensive and assertive services [7, 10]. Some of these patients may have limited contact with services [7] with few or no hospital admissions. In this scenario, hospitalization may help stabilize a difficult situation that might easily be overlooked by traditional, office-based mental health services. With the growing focus on recovery-oriented practices in ACT [8], these teams may offer benefits for patients with severe mental illness and high needs, even if their problems have not led to high use of inpatient services.

Identification of differences between high users and low users of inpatient care and factors associated with changes in hospitalization is therefore important to increase the understanding of the impact that ACT may have on these subgroups. However, different criteria have been used to define high use of inpatient care, either based on the number of admissions [13, 16, 17] or total inpatient days over a fixed time period [14, 18]. Definitions using only frequency exclude patients with few but long admissions while those using only duration exclude patients with frequent but short admissions. To our knowledge, the REACT study from the UK is the only ACT trial that applied criteria that accounted for both frequency and duration [19].

The ACT model was recently introduced to Norway to improve services to patients with severe mental illness (schizophrenia, other psychotic disorders or severe bipolar disorder) who were difficult to reach and engage by existing services. The Norwegian mental health service system is divided into two organizational levels, with primary health and social care provided at the municipal level and specialized mental health services provided by state-owned health authorities. The primary mental health care comprises general practitioners, individual or group therapy, self-help groups, day centers, and supported housing with full or partial supervision. The specialized mental health services comprise community mental health centers (CMHCs) and psychiatric departments in hospitals. The CMHCs comprise outpatient clinics, psychosis rehabilitation teams, substance abuse clinics day units, crisis resolution teams, and local inpatient facilities.

The key principles of ACT, including outreach, delivery of services in the community, holistic and integrated services, and continuity of care [20] may have been incorporated in standard mental health care internationally but this is not the case in many mental health care settings in Norway. The services are often fragmented and office-based, and the complexity of the service configuration may present impediments to access appropriate treatment for people with severe mental illness.

Estimates from 2008 suggested that more than 4000 persons with severe mental illness in Norway (approximately 1/1000 inhabitants) did not receive appropriate mental health services [21]. In 2009, the National Health Authorities decided to fund implementation of ACT teams across Norway to improve services for this population. Between December 2009 and February 2011, 12 ACT teams were established throughout the country. A history of high inpatient service use was not an inclusion criterion, and this provided an opportunity to investigate possible differences between high users and low users of hospitalization, applying the criteria used in the REACT study. Based on the existing ACT literature, we expect that high users would experience a decrease in hospitalization during ACT follow-up while hospitalization would increase among the low users.

### Aims and research questions

This study aimed to investigate hospitalization (new admissions, total inpatient days, involuntary inpatient days) amongst high and low inpatient service users in the 2 years before and 2 years after enrollment into Norwegian ACT teams, and to explore factors associated with change in hospitalization. Our specific research questions were: are there significant socio-demographic or clinical differences between high users and low users of inpatient care upon ACT enrollment? Are there differences in hospitalization in the 2 years before ACT compared to the 2 years during ACT in the two groups? Are changes in hospitalization in the 2 years before ACT compared to the 2 years after enrollment associated with patient characteristics upon enrollment?

## Methods

### Design

This paper is based on data from the naturalistic observational study on ACT teams in Norway. Cross-sectional socio-demographic and clinical data from 142 patients of 12 ACT teams upon enrollment and longitudinal hospitalization data in the 2 years before and 2 years after ACT enrollment were used in this paper. Due to the nature of the funding and the implementation of the ACT model in Norway, it was not possible to conduct a randomized trial. However, a naturalistic observational study was designed to investigate patient outcomes in a real-life, clinical world.

### Recruitment and sample

The ACT teams used inclusion criteria defined by the National Health Authorities which are similar to criteria used in international ACT studies: 18 years or older; severe mental illness (schizophrenia, schizoaffective, other psychotic disorder, bipolar affective disorder); impaired level of functioning; in need of long-term and comprehensive follow-up by mental health and social welfare services.

Patients with co-occurring substance misuse were included if this was not the primary diagnosis.

During the ACT teams’ first year of operation 337 patients enrolled in the 12 teams and they were all invited to participate in the study. A total of 202 patients (60 %) gave written informed consent to participate after the procedure was fully explained. Of these, 142 (42 %) received ACT services for at least 2 years, and were considered eligible for this study (participants n = 142). Data on inpatient service use was not available for the non-participants (n = 195).

Compared to the non-participants, fewer participants had problematic substance misuse (n = 83 versus 128, 59 % versus 70 %, p = 0.034). Participants had less severe symptoms (mean score ± standard deviation (SD) Global Assessment of Functioning-Symptom Scale (GAF-S), 41.4 ± 10.2 versus 38.8 ± 10.0, p = 0.028) and better functioning (mean score ± SD Global Assessment of Functioning-Function Scale (GAF-F), 39.7 ± 8.3 versus 37.6 ± 8.9, p = 0.036). There were no differences in age, gender, diagnosis of severe mental illness, or number of people subject to involuntary outpatient treatment.

The classification of high use of inpatient services prior to ACT were based on the inclusion criteria applied in the REACT study [19]: five or more psychiatric admissions in mental health hospitals or at least 100 consecutive inpatient days during the last 2 years, or three or more admissions or at least 50 consecutive inpatient days during the last year [19]. Of the 142 participants, 74 (52 %) were high users of inpatient services prior to ACT and 68 (48 %) were not.

### Measures

#### Clinician-rated instruments

Socio-demographic data were collected using a form developed by the research group. Global level of functioning was assessed with the Global Assessment of Functioning (GAF) scale [22]. Psychiatric symptoms were assessed with the expanded version of the Brief Psychiatric Rating Scale (BPRS, version 4) [23, 24]. The BPRS-4 comprises 24 items, giving four subscales (i.e., positive symptoms, negative symptoms, agitation mania, and anxiety/depressive symptoms) [25]. Each item is given a score from 1 (not present) to 7 (extremely severe). Everyday functioning was measured with the revised version of the Practical and Social Functioning Scale (PSF) [26], consisting of 32 items. The mean total score ranges from 0 to 8, where higher scores indicate better functioning. An adapted version of the Homeless Engagement and Acceptance Scale (HEAS) [27] measured participants’ quality of engagement with services. The HEAS consists of four items, three rated from 0 to 4 and one from 0 to 3, giving a total score between 0 and 15. Higher scores indicate better service engagement.

#### Self-reported questionnaires

The alcohol use disorder identification test (AUDIT) [28] and the drug use disorder identification test (DUDIT) [29] are self-report instruments that screen for problematic substance use in the last 12 months. The AUDIT comprises ten and the DUDIT comprises eleven items, with total scores ranging from 0 to 40 (AUDIT) and 0 to 44 (DUDIT). Scores above specific cut-offs (AUDIT: men 8, women 6; DUDIT: men 6, women 2) indicate problematic substance use and higher score indicates greater severity.

### Data-collection

Data on number of new admissions, total and involuntary inpatient days in mental health hospitals for the 142 participants in the 2 years before and the 2 years after enrollment into ACT was obtained from the Norwegian Patient Registry. Socio-demographic and clinical data were collected by the ACT teams when the participants enrolled into the teams. Both clinician-rated and self-reported questionnaires were used. Information was obtained through interviews with patients, care givers, and professionals, from direct observations and case-note reviews. The self-reported questionnaires were filled in by the participants alone or together with a team member.

### Fidelity of Norwegian ACT teams

The Norwegian teams’ fidelity to the ACT model was assessed using the Tool for Measurement of Assertive Community Treatment (TMACT) [8]. The TMACT comprises 47 items, giving six subscales; organization and structure (OS), core team (CT, including team leader, nursing staff and psychiatric care provider), specialist team (ST, including substance abuse specialist, vocational specialist, and peer specialist), core practices (CP), evidence-based practices (EP) and person-centered planning and practices (PP). Each of the 47 items is rated on a 5-point scale from 1 (not implemented) to 5 (fully implemented). The fidelity was measured at 12 and 30 months after the teams were established. The mean TMACT scores at 12 months ranged from 2.7 to 3.7, indicating low to moderate fidelity and at 30 months the scores ranged from 3.1 to 4.1, indicating moderate to high fidelity. At 30 months, the mean scores on the different subscales showed low implementation on ST, moderate fidelity on CP, EP and PP, and high implementation on OS and CT.

### Statistical analysis

Differences in socio-demographic and clinical characteristics between high and low users were assessed with Fisher’s exact test for dichotomous variables, Chi square test for categorical variables, Student’s *T* test for symmetrically distributed continuous variables, and Mann-Whitney U test for skewed continuous variables.

Total and involuntary inpatient days for four periods [time period (TP) 1: 24–12 months pre-ACT enrollment and TP2: 12–0 months pre-enrollment, TP3: 0–12 months post-enrollment and TP4: 12–24 months post-enrollment] were presented as means and 95 % confidence intervals (CI).

To assess changes in hospitalization the difference between the number of *new admissions, total inpatient days* and *involuntary inpatient days* in the 2 years before and the 2 years after ACT enrollment were defined as dependent variables.

The level of clustering within the team was assessed by an intra-class coefficient (ICC). Only a weak cluster effect was present but nevertheless, the difference in hospitalization between high and low users was analyzed by a linear mixed model with random effects at the ACT level, to correctly adjust the estimates for possible intra-ACT correlations. Fixed effect for variable identifying high and low users was entered into the model.

A multivariate linear mixed model was built with clinical variables [involuntary outpatient treatment (Y/N), the four BPRS subscales, AUDIT, DUDIT, HEAS, PSF, and high inpatient service use (Y/N)] as fixed effects to assess possible predictors for change in hospitalization. Random effects at the ACT level were included. The Akaike’s information criteria (AIC) (the smaller the better) [30] was applied for model reduction, but according to the AIC, none of the predictors could be eliminated. The final model was adjusted for age, gender and fidelity score (TMACT mean score at 30 months as this score was thought to best represent the 2 year follow-up period of the participants).

We imputed missing values on PSF items (n = 14, 0.3 % of cases), HEAS items (n = 2, 0.4 %), AUDIT (n = 14, 9.9 %) and DUDIT (n = 18, 12.7 %) by generating the empirical distribution for each variable and drawing a random number from that distribution to replace the missing value. The process was repeated until all missing values were imputed. The GAF scores were close to normally distributed, and missing values (n = 4, 2.8 % of cases) were imputed by drawing a random number from the corresponding normal distribution. The BPRS was completed for 98.6 % of the participants and thus we imputed no scores.

Linear mixed models were estimated by Statistical Analysis System version 9.3 (SAS Institute, Cary, NC USA). Other statistical analyses were performed with the Statistical Package for Social Science version 22 (SPSS, Chicago, IL USA). All tests were two-sided. p values below 0.05 were considered statistically significant. No correction for multiple hypothesis testing was performed as the study was exploratory.

### Ethics, consent and permission

The study was approved by the Regional Committee for Medical and Health Research Ethics Health region South-East (ID: 2010/1196a). All participants gave written informed consent to participate in the study after the procedure of the study had been explained to them by the ACT teams.

## Results

### Characteristics of the groups

Upon ACT enrollment, the high users were younger, more likely to be subject to involuntary outpatient treatment, more likely to live in supported accommodations, be in prison or homeless, and less likely to live alone, as compared to the low users (Table 1). There were no significant differences in scores on clinical rating assessments between the groups.

**Table 1:** Socio-demographic and clinical characteristics of high and low users upon ACT enrollment

	Non-high users		High users		p value
	N	%	N	%	
Socio-demographic characteristics					
Sex (male)	43	64	51	69	0.594^a^
Age, mean (SD)	*42 (10.8)*		*38 (9.7)*		*0.015* ^c^
Ethnicity					0.890^b^
Norwegian	54	83	60	85	
Other European	6	9	5	7	
Outside Europe	5	8	6	8	
Marital status					0.710^b^
Unmarried	49	72	57	77	
Married/cohabitant	7	10	5	7	
Education					0.243^b^
Completed primary school	36	58	40	57	
Completed upper secondary school	23	37	21	30	
Completed higher education	3	5	9	13	
Employment status					0.669^b^
Unemployed	56	83	62	84	
Competitive job/study	5	7	3	4	
Other	7	10	9	12	
Living situation					*0.034* ^b^
Alone	49	72	42	57	
With family	15	22	16	22	
Staffed housing/supported housing/institutions (hospital, prison, hospice)/homeless/unstable living situation	4	6	15	21	
Clinical Characteristics					
Diagnosis					0.659^b^
Schizophrenia, schizo-affective or other psychotic disorder	53	86	62	89	
Bipolar disorder	4	6	5	7	
Other psychiatric disorder	5	8	3	4	
Community treatment order (yes)	14	21	37	50	<*0.001* ^a^
Substance abuse					0.321^b^
None	32	47	27	37	
Alcohol (AUDIT)	12	18	10	13	
Other substances (DUDIT)	11	16	14	19	
Alcohol and other substances (AUDIT and DUDIT)	13	19	23	31	

	Mean	SD	Mean	SD	p value
AUDIT total score	7.87	9.21	8.30	9.05	0.954^d^
DUDIT total score	7.54	11.37	10.58	13.01	0.141^d^
Psychiatric symptoms					
BPRS mean total score	2.51	0.82	2.40	0.79	0.408^d^
BPRS positive symptoms	2.50	1.32	2.47	1.23	0.938^d^
BPRS negative symptoms	2.60	1.24	2.39	1.07	0.316^d^
BPRS agitation mania	2.25	1.16	2.07	1.02	0.369^d^
BPRS anxiety/depressive symptoms	2.80	0.89	2.64	1.11	0.362^c^
Global level of functioning (GAF)	38.6	8.7	37.4	8.6	0.412^c^
Level of functioning (PSF)	4.33	4.33	4.25	1.58	0.781^c^
Engagement and acceptance of contact with services (HEAS)	9.42	3.00	9.89	2.97	0.360^c^

^a^ Fischer’s exact test

^b^ Chi square

^c^ Student’s *T* test

^d^ Mann–Whitney U Test

### Hospitalization

There were few differences between the 12 ACT teams regarding patients’ inpatient service use before ACT [total inpatient days (ICC = 7.4 %), involuntary inpatient days (ICC = 6.2 %)]. There were also only small differences between the teams regarding change in total inpatient days (ICC = 2.8 %) and involuntary days (ICC = 1.1 %).

For the total sample, the mean number of new admissions was the same before and after ACT enrollment; on average, patients had three admissions in the 2 years before and three admissions in the 2 years after enrollment (Table 2). However, both total and involuntary inpatient days were halved in the 2 years after ACT enrollment compared to the 2 years before. There were significant differences in the changes in inpatient service use between the high and the low users. Total and involuntary inpatient days reduced amongst the high users, whilst the low users experienced an increase in the same period.

**Table 2:** Hospitalization of total population, high and low users two years before and during ACT

Outcome	Population	Before ACT enrollment		After ACT enrollment		Change before-after ACT enrollment		
		Mean	SD	Mean	SD	Mean^a^	95 % CI	p value^b^
New admissions	Total population^c^	3.34	3.98	3.00	4.70	0.27	−0.49 to 1.04	0.480
	Low users^d^	1.28	1.23	1.62	3.02	−0.33	−0.96 to 0.29	0.284
	High users^e^	5.23	4.64	4.39	5.56	0.84	−0.52 to 2.20	0.223
Total inpatient days	Total population^c^	120.93	154.63	61.47	77.58	59.46	32.88 to 86.03	<*0.001*
	Low users^d^	26.57	31.37	50.94	82.82	−24.37	−43.37 to −5.37	*0.013*
	High users^e^	207.64	171.37	71.15	71.64	136.49	95.47 to 177.51	<*0.001*
Involuntary inpatient days	Total population^c^	80.82	147.45	36.63	64.99	44.20	19.30 to 69.10	<*0.001*
	Low users^d^	11.76	22.44	31.43	70.54	−19.66	−36.42 to −2.91	*0.022*
	High users^e^	144.28	181.68	41.41	59.51	102.88	61.55 to 144.20	<*0.001*

^a^ Positive results indicate mean reduction in outcome after ACT enrollment compared to before while negative results indicate mean increase

^b^ Analyses of changes using linear mixed models

^c^ Total population N = 142

^d^ Low users N = 68

^e^ High users N = 74

When comparing the three hospitalization outcomes in the four time periods (TP1, TP2, TP3, TP4, Table 3), the high users experienced an increase in all outcomes before ACT (TP1–TP2) and a decrease after ACT enrollment (TP2–TP4). However, the low users experienced an increase in new admissions throughout the period (TP1–TP4). Total and involuntary inpatient days were stable before ACT amongst the low users (TP1–TP2) but both outcomes increased in the first year after ACT enrollment (TP2–TP3) and subsequently decreased during the second year (TP 3–TP4). Non-overlapping confidence intervals between to consecutive periods indicate significant change between these two periods.

**Table 3:** Hospitalization of total population, high and low users (four time periods)

Outcome	Population	TP 1^a^		TP 2^b^		TP 3^c^		TP 4^d^	
		Mean	95 % CI	Mean	95 % CI	Mean	95 % CI	Mean	95 % CI
New admissions	Total population	1.39	1.05–1.74	1.94	1.55–2.34	1.61	1.23–1.99	1.45	0.99–1.91
	Low users	0.57	0.35–0.79	0.71	0.51–0.9	0.79	0.52–1.06	0.82	0.28–1.36
	High users	2.15	1.56–2.74	3.08	2.45–3.71	2.36	1.71–3.02	2.03	1.31–2.75
Total inpatient days	Total population	51.08	36.63–65.52	69.85	55.20–84.50	36.61	28.50–44.73	24.86	16.37–33.53
	Low users	12.63	7.23–18.04	13.94	9.24–18.65	29.41	17.59–41.24	21.53	6.93–36.13
	High users	86.41	61.51–111.3	121.23	99.09–143.37	43.23	32.02–54.44	27.92	18.36–37.47
Involuntary inpatient days	Total population	33.48	20.97–45.99	47.35	32.94–61.75	23.83	16.33–31.33	12.80	6.37–19.22
	Low users	6.72	2.36–11.08	5.04	2.26–7.83	21.81	10.34–33.28	9.62	−0.23–19.46
	High users	58.07	35.59–80.54	86.22	61.66–110.78	25.69	15.63–35.74	15.72	7.17–24.26

Not adjusted for ACT level

^a^TP1 = 24–12 months before ACT enrollment

^b^ TP2 = 12–0 months before ACT enrollment

^c^ TP3 = 0–12 months after ACT enrollment

^d^ TP4 = 12–24 months after ACT enrollment

### Patient characteristics associated with changes in inpatient days

The exploratory regression analyses showed that fidelity was not associated with changes in new admissions, total inpatient days or involuntary days. There were also no significant associations between change in new admissions and patient characteristics. However, more severe negative symptoms and high use of inpatient services before ACT were significantly associated with reduction in both total and involuntary inpatient days after ACT enrollment (Table 4). Being subject to involuntary outpatient treatment upon enrollment was also significantly associated with a reduction in involuntary inpatient days after ACT enrollment.

**Table 4:** Multivariate linear mixed models: Associations between patient characteristics and change in total and involuntary inpatient days (N = 124)

Variables	Total inpatient days		Involuntary inpatient days	
	Regression coefficient (95 % CI)	p value	Regression coefficient (95 % CI)	p value
Age	37.88 (−15.72; 91.48)	0.164	43.35 (−9.55; 96.26)	0.107
Gender	−1.31 (−4.10; 1.49)	0.357	0.29 (−2.47; 3.05)	0.836
Involuntary outpatient treatment	28.37 (−29.86; 86.61)	0.336	62.93 (5.46; 120.41)	*0.032*
BPRS positive symptoms	−0.77 (−26.65; 25.10)	0.953	−4.49 (−30.02; 21.05)	0.728
BPRS negative symptoms	36.23 (11.24; 61.23)	*0.005*	31.95 (7.28; 56.61)	*0.012*
BPRS agitation mania	−16.82 (−49.03; 15.39)	0.303	−19.58 (−51.36; 12.21)	0.225
BPRS anxiety/depressive symptoms	−17.05 (−43.19; 9.09)	0.199	−13.66 (−39.46; 12.14)	0.296
AUDIT	0.69 (−2.43; 3.81)	0.663	−0.06 (−3.14; 3.02)	0.969
DUDIT	1.29 (−1.05; 3.63)	0.277	2.07 (−0.23; 4.38)	0.078
HEAS	7.71 (−1.72; 17.15)	0.108	7.73 (−1.58; 17.04)	0.103
PSF	15.57 (−3.20; 34.34)	0.103	9.29 (−9.23; 27.81)	0.323
High frequency user	141.14 (88.26; 194.02)	<*0.001*	95.52 (43.33; 147.71)	<*0.001*
TMACT mean score 30 months	32.31 (−66.15; 130.76)	0.517	45.39 (−51.78; 142.57)	0.357

## Discussion

Our study documented a decrease in total and involuntary inpatients days over the 2 years of ACT follow-up but no change in number of admissions. The decrease in inpatient days was more evident for the high users whilst for the low users there was an initial increase and a subsequent decrease in inpatient days after ACT enrollment. More severe negative symptoms upon ACT enrollment and high inpatient service use before ACT were significantly associated with a reduction in both total and involuntary inpatient days after ACT enrollment. Additionally, a reduction in involuntary inpatient days was significantly associated with being subject to involuntary outpatient treatment upon ACT enrollment.

### Characteristics of the groups

Our findings that the high users were younger, more often subject to involuntary outpatient treatment and less likely to be living independently compared to the low users upon ACT enrollment is corroborated by previous studies [16, 17]. The fact that there were no differences in ratings of clinical problems between the groups may support the hypothesis of Mortimer and colleagues that ACT could be appropriate for patients with severe mental illness who are not high users of inpatient services [15]. According to the NICE guidelines, in addition to reducing the use of hospitalization, ACT teams should ensure continuous contact with services and improve psychosocial outcomes [3]. Intensive case management, including ACT, has been shown to have a significant advantage over other services in reducing the number of people who drop-out of contact with services [6]. It may be that the increase in hospitalization experienced by the low users represented an appropriate response to unmet clinical needs, or it may have shown a negative impact of ACT involvement. The fact that inpatient days reduced in the second year of ACT in this group perhaps gives more weight to the first explanation, suggesting that admission was necessary to attend to specific problems in order that the person could progress. Additionally, a recovery approach is an important part of ACT [14] and ACT may therefore provide a basis for recovery-oriented, assertive, and intensive services to patients with significant clinical needs who historically have not been high users of inpatient services.

### Hospitalization

We found that the participants spent significantly fewer days in hospital during the 2 years of ACT follow-up compared to the 2 years before they enrolled into the teams. This is in contrast to findings from recent European randomized trials of ACT [19, 31], but is in line with several non-randomized studies [14, 15, 32, 33]. That the reduction in inpatient days was mainly found amongst the high users, supporting findings by Burns and colleagues [12] and Dietrich and colleagues [6].

Previous studies have suggested that ACT has most impact on hospitalization where there is less overlap with standard care services [11, 12]. In England, Crisis Resolution Teams (CRTs) and ACT teams were implemented simultaneously as part of a national policy and the subsequent reduction in use of hospitalization was attributed to the CRTs more than ACT [34], although this finding has been questioned [35]. In contrast, the CRTs in Norway were established before the ACT teams and serve a population with less severe symptoms [36]. This suggests that the presence of CRTs and ACT in the same catchment area is an unlikely explanation for the reduction in hospitalization found in our study.

When inpatient service use is already low, the effect of interventions aiming to reduce hospitalization is less likely to succeed [12, 37]. National data from 2009 [38] and 2013 [39] show that high users of inpatient services, of whom the majority suffer severe mental illness like schizophrenia, spend an average of 75–83 days in hospital per year. This is similar to the level of total inpatient days per year we found in our study in the 2 years before ACT but it is almost twice as high as the number of total inpatient days per year in the 2 years of ACT follow-up. This may indicate that, although the design of our study does not allow us to draw conclusions regarding the effect of ACT on hospitalization, it is unlikely that regression to the mean can fully explain the reduction found amongst high users in our study.

However, national policies on hospital bed availability can also influence use of inpatient services. Between 2009 and 2013 there was a 13 % reduction in the number of inpatient beds, a 15.3 % reduction in total inpatient days [39], and minor fluctuations in the use of involuntary inpatient treatment in Norway [40]. Although these figures include all patients, not only those with severe mental illness, they are unlikely to support the possibility that changes in inpatient services explain the much larger reduction in inpatient service use found in this study (reduction in total inpatient days for all participants 59.46 days, p < 0.001, reduction in involuntary inpatient days for all participants 44.20 days, p < 0.001). Furthermore, although total inpatient days were reduced during ACT follow-up, there was no increase in involuntary inpatient treatment. This could indicate that patients experiencing deterioration were identified at an earlier stage of relapse by ACT, prior to requiring involuntary admission.

### Patient characteristics associated with changes in inpatient days

An increase in total inpatient days during ACT among patients with low baseline use has previously been reported [13, 14] and is not surprising, as reduction in total hospitalization is primarily found if baseline use is high [12]. High use of inpatient services and involuntary outpatient treatment were both associated with reduced inpatient service use, as was having more severe negative symptoms upon ACT enrollment. Although exploratory, our findings may support the hypothesis that hospitalization during ACT can mark the beginning of access to care and recovery for patients with prior low inpatient service use [13], and that ACT may contribute to more appropriate use of inpatient services and involuntary hospitalization amongst patient both with and without high inpatient service use.

Our study showed no associations between the teams’ fidelity score at 30 months and changes in hospitalization after ACT enrollment, in contrast to recent reports that higher TMACT scores were associated with decreased hospital use [41]. However, our study was exploratory so our findings should be interpreted with caution.

### Strengths and limitations

A major strength of our study is that we have data from 12 different ACT teams operating in both urban and rural areas, covering all parts of Norway. Instruments with good psychometric properties were used. However, our study is an observational study and not a randomized controlled trial and therefore subject to potential confounders. We cannot conclude that the reduction in hospitalization observed was due to ACT, although the reduction was similar across teams and much higher than the national reduction in inpatient service use. Our sample included only those who gave informed consent and had received ACT for at least 2 years in Norway. Therefore our results may not be generalizable to all ACT patients. Finally, there were fewer participants than non-participants with substance abuse and the participants had statistically better functioning and less severe symptoms. This may have contributed to an overestimation of the reduction in total and involuntary inpatient days found in this study although the difference in symptom and functioning levels between the groups may not have been clinically significant.

## Conclusion

This study showed a clear reduction in both total and involuntary inpatient days after the patients enrolled into ACT. The reduction was mainly due to fewer inpatient days amongst the high users. The low users experienced an initial increase in inpatient days, perhaps required to attend to needs that had not been identified by other services. Our results suggest that ACT may contribute to a more appropriate use of inpatient care for both groups, possibly by reducing the presumably avoidable hospitalization of high users and increasing the presumably undetected but needed inpatient care by the low users.

## Abbreviations

ACT: Assertive Community Treatment
CMHC: Community Mental Health Centers
GAF: Global Assessment of Functioning
BPRS: Brief Psychiatric Rating Scale
PSF: practical and social functioning
HEAS: Homeless Engagement and Acceptance Scale
AUDIT: Alcohol Use Disorder Identification Scale
DUDIT: Drug Use Disorder Identification Scale
TMACT: tool for measurement of Assertive Community Treatment
OS: organization and structure
CT: Core Team
ST: Specialist Team
CP: core practices
EP: evidence-based practices
PP: person-centered planning and practices
TP: time period
CI: confidence interval
ICC: intra-class correlation coefficient
AIC: Akaike’s information criteria
SAS: statistical analysis system
SPSS: statistical package for social science
CRT: Crisis Resolution Team
SD: standard deviation
